# Penta­carbonyl-2κ^5^ *C*-chlorido-1κ*Cl*-bis­[1(η^5^)-cyclo­penta­dien­yl][μ-oxido(phenyl)methylene-1:2κ^2^ *O*:*C*]hafnium(IV)tungsten(0)

**DOI:** 10.1107/S1600536808025245

**Published:** 2008-08-09

**Authors:** Catharine Esterhuysen, I. B. Jacques Nel, Stephanie Cronje

**Affiliations:** a Department of Chemistry and Polymer Science, University of Stellenbosch, Private Bag X1, Matieland 7602, South Africa

## Abstract

The title compound, [HfW(C_5_H_5_)_2_(C_7_H_5_O)Cl(CO)_5_] or [W(CO)_5_(C_7_H_5_O){Hf(C_5_H_5_)_2_Cl}], contains two metal centres, with a (tungstenpenta­carbon­yl)oxy­phenyl­carbene unit coordinated to a hafnocene chloride. The Hf—O—C angle is nearly linear, and the C=O distance is slightly shorter than for equivalent alkoxy­carbenes. One of the cyclo­penta­dienyl (Cp) rings undergoes an offset face-to-face π–π inter­action [3.495 (7) Å] with the symmetry-related Cp ring of a neighbouring mol­ecule.

## Related literature

For related literature regarding anionic Fischer-type carbenes, see: Barluenga & Fañanás (2000 ▶); Brüll *et al.* (2001 ▶). For comparable structures, see: Berlekamp *et al.* (1993 ▶); Erker *et al.* (1989 ▶, 1991 ▶). For comparable bond lengths, see: Orpen *et al.* (1989 ▶).
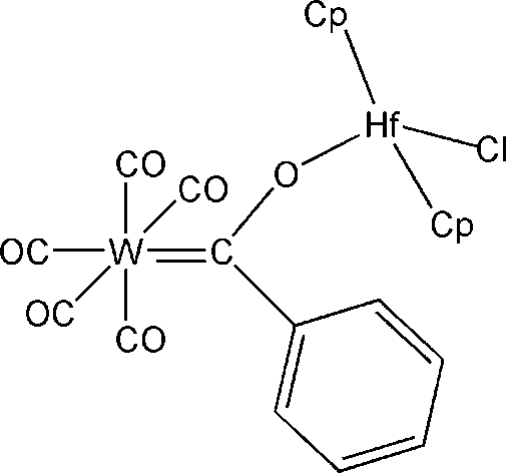


## Experimental

### 

#### Crystal data

[HfW(C_5_H_5_)_2_(C_7_H_5_O)Cl(CO)_5_]
*M* *_r_* = 773.13
Monoclinic, 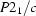

*a* = 8.5422 (2) Å
*b* = 12.5546 (3) Å
*c* = 21.0237 (7) Å
β = 96.152 (1)°
*V* = 2241.68 (11) Å^3^
*Z* = 4
Mo *K*α radiation
μ = 9.91 mm^−1^
*T* = 173 (2) K
0.33 × 0.27 × 0.25 mm

#### Data collection

Nonius KappaCCD diffractometer
Absorption correction: multi-scan (*DENZO-SMN*; Otwinowski & Minor, 1997 ▶) *T*_min_ = 0.056, *T*_max_ = 0.089 (expected range = 0.053–0.084)
12410 measured reflections
5106 independent reflections
4234 reflections with *I* > 2σ(*I*)
*R*_int_ = 0.049

#### Refinement

*R*[*F* ^2^ > 2σ(*F* ^2^)] = 0.032
*wR*(*F* ^2^) = 0.079
*S* = 1.01
5106 reflections
280 parameters
H-atom parameters constrained
Δρ_max_ = 2.61 e Å^−3^
Δρ_min_ = −1.76 e Å^−3^

### 

Data collection: *COLLECT* (Nonius, 1998 ▶); cell refinement: *DENZO-SMN* (Otwinowski & Minor, 1997 ▶); data reduction: *DENZO-SMN*; program(s) used to solve structure: *SHELXS97* (Sheldrick, 2008 ▶); program(s) used to refine structure: *SHELXL97* (Sheldrick, 2008 ▶); molecular graphics: *X-SEED* (Barbour, 2001 ▶; Atwood & Barbour, 2003 ▶); software used to prepare material for publication: *publCIF* (Westrip, 2008 ▶).

## Supplementary Material

Crystal structure: contains datablocks I, global. DOI: 10.1107/S1600536808025245/tk2291sup1.cif

Structure factors: contains datablocks I. DOI: 10.1107/S1600536808025245/tk2291Isup2.hkl

Additional supplementary materials:  crystallographic information; 3D view; checkCIF report

## Figures and Tables

**Fig. 1.**
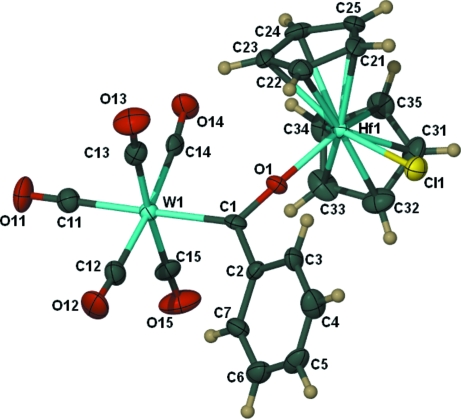
The molecular structure of I showing the atomic labelling scheme and displacement ellipsoids drawn at the 50% probability level.

## supplementary crystallographic information

### Comment

Anionic Fischer-type carbene ligands, prepared by the standard addition of organolithium compounds to metal carbonyls, act as monodentate ligands towards transition metals like Ti and Zr (Barluenga and Fañanás, 2000). When the second metal unit is a zirconocene moiety, Cp_2_ZrCl, then such complexes have been proven to catalyse the oligomerization of 1-pentene in the presence of methylaluminoxane, MAO (Brüll *et al.*, 2001). Herein, we report the Hf equivalent of these zirconocene alkoxycarbene complexes.

In the title compound, (I, Fig. 1), the W=C_carbene_ and C_carbene_—C distances are similar to those found in alkoxycarbene complexes, whereas the C—O distance is shorter [2.16 (1), 1.50 (3) and 1.47 (2) Å, respectively; Orpen *et al.*, 1989]. The Hf—O distance is also shorter than those in the metallocyclic compounds C_26_H_27_HfO_5_V [2.063 (3) Å; Erker *et al.*, 1991] and C_28_H_29_HfO_5_V [2.066 (3) Å; Berlekamp *et al.*, 1993]. The Hf—O—C angle is nearly linear, with a larger value [171.4 (3)°] than the equivalent Hf—O—C angles of 163.6 (3) and 169.0 (3)° in C_26_H_27_HfO_5_V (Erker *et al.*, 1991) and C_28_H_29_HfO_5_V (Berlekamp *et al.*, 1993), respectively, as well as the Zr—O—C angle of 166.1 (5)° in W(CO)_5_C(C_6_H_5_)OZr(C_5_H_5_)_2_OC_6_H_5_ (Erker *et al.*, 1989).

The C21/C22/C23/C24/C25 Cp ring [with centroid *Cg*(1)] undergoes offset face-to-face π–π interactions with the symmetry related Cp ring on a neighbouring molecule [*Cg*(1)···*Cg*(1)^i^ = 3.495 (7) Å; Symmetry code: (i) 1 - *x*, 2 - *y*, 1 - *z*)].

### Experimental

A solution of LiCH_3_ (31 ml, 1.6*M*) in diethylether (50 ml) was added to a well stirred suspension of W(CO)_6_ (17.802 g) in diethylether (100 ml). After solvent removal, dissolution of the residue in cold water (150 ml) and filtration, a solution of Et_4_NCl (8.721 g) in cold water (50 ml) was added to the filtrate. Upon further filtration 0.740 g of the product {[W(CO)_5_C(C_6_H_5_)O][NEt_4_]} was dissolved in dichloromethane (70 ml) and added to a solution of Cp_2_HfCl_2_ (0.505 g) in dichloromethane (40 ml). After stirring for 30 min at -40°C AgBF_4_ (0.261 g) was added. The solvent was removed and the residue extracted in 5 portions of 10 ml toluene. The extract was cooled to -40°C and filtered. The filtrate was dried over anhydrous MgSO_4_, concentrated to saturation, and kept at -6°C, whereupon red crystals of the title compound suitable for X-ray diffraction analysis were obtained in 19% yield.

### Refinement

H atoms were positioned geometrically with C—H = 0.95 Å and constrained to ride on their parent atoms with *U*_iso_(H) = 1.2*U*_eq_(C). The maximum and minimum residual electron density peaks were located 0.93 and 0.83 Å, respectively from the Hf1 and W1 atoms.

### Crystal data

**Table:**

[HfW(C_5_H_5_)_2_(C_7_H_5_O)Cl(CO)_5_]	*F*_000_ = 1432
*M**_r_* = 773.13	*D*_x_ = 2.291 Mg m^−^^3^
Monoclinic, *P*2_1_/*c*	Mo *K*α radiation λ = 0.71073 Å
Hall symbol: -P 2ybc	Cell parameters from 12410 reflections
*a* = 8.5422 (2) Å	θ = 1.9–27.5º
*b* = 12.5546 (3) Å	µ = 9.91 mm^−^^1^
*c* = 21.0237 (7) Å	*T* = 173 (2) K
β = 96.152 (1)º	Prism, red
*V* = 2241.68 (11) Å^3^	0.33 × 0.27 × 0.25 mm
*Z* = 4	

### Data collection

**Table:**

Nonius KappaCCD diffractometer	5106 independent reflections
Radiation source: fine-focus sealed tube	4234 reflections with *I* > 2σ(*I*)
Monochromator: graphite	*R*_int_ = 0.049
*T* = 173(2) K	θ_max_ = 27.5º
φ and ω scans to fill Ewald sphere	θ_min_ = 1.9º
Absorption correction: multi-scan(DENZO-SMN; Otwinowski & Minor, 1997)	*h* = −11→8
*T*_min_ = 0.056, *T*_max_ = 0.089	*k* = −16→15
12410 measured reflections	*l* = −26→27

### Refinement

**Table:**

Refinement on *F*^2^	Secondary atom site location: difference Fourier map
Least-squares matrix: full	Hydrogen site location: inferred from neighbouring sites
*R*[*F*^2^ > 2σ(*F*^2^)] = 0.032	H-atom parameters constrained
*wR*(*F*^2^) = 0.079	*w* = 1/[σ^2^(*F*_o_^2^) + (0.0436*P*)^2^] where *P* = (*F*_o_^2^ + 2*F*_c_^2^)/3
*S* = 1.01	(Δ/σ)_max_ = 0.002
5106 reflections	Δρ_max_ = 2.61 e Å^−^^3^
280 parameters	Δρ_min_ = −1.76 e Å^−^^3^
Primary atom site location: heavy-atom method	Extinction correction: none

### Special details

**Table:**

Geometry. All e.s.d.'s (except the e.s.d. in the dihedral angle between two l.s. planes) are estimated using the full covariance matrix. The cell e.s.d.'s are taken into account individually in the estimation of e.s.d.'s in distances, angles and torsion angles; correlations between e.s.d.'s in cell parameters are only used when they are defined by crystal symmetry. An approximate (isotropic) treatment of cell e.s.d.'s is used for estimating e.s.d.'s involving l.s. planes.
Refinement. Refinement of *F*^2^ against ALL reflections. The weighted *R*-factor *wR* and goodness of fit *S* are based on *F*^2^, conventional *R*-factors *R* are based on *F*, with *F* set to zero for negative *F*^2^. The threshold expression of *F*^2^ > σ(*F*^2^) is used only for calculating *R*-factors(gt) *etc*. and is not relevant to the choice of reflections for refinement. *R*-factors based on *F*^2^ are statistically about twice as large as those based on *F*, and *R*- factors based on ALL data will be even larger.

### Fractional atomic coordinates and isotropic or equivalent isotropic displacement parameters (Å^2^)

**Table:**

	*x*	*y*	*z*	*U*_iso_*/*U*_eq_	
Hf1	0.80934 (3)	0.779762 (17)	0.542399 (9)	0.01909 (8)	
W1	0.46096 (3)	0.614746 (17)	0.676791 (9)	0.02237 (8)	
Cl1	1.06982 (17)	0.83902 (12)	0.58304 (7)	0.0346 (3)	
O1	0.3696 (5)	0.6436 (4)	0.52755 (18)	0.0401 (11)	
O2	0.1151 (5)	0.5226 (4)	0.6774 (2)	0.0515 (12)	
O3	0.4999 (6)	0.6018 (4)	0.82830 (19)	0.0530 (13)	
O4	0.3679 (6)	0.8575 (4)	0.6959 (2)	0.0525 (13)	
O5	0.5903 (7)	0.3811 (4)	0.6553 (2)	0.0590 (15)	
O6	0.7432 (5)	0.7258 (3)	0.62527 (15)	0.0235 (8)	
C1	0.4097 (6)	0.6334 (4)	0.5806 (3)	0.0250 (12)	
C2	0.2402 (7)	0.5579 (5)	0.6782 (3)	0.0324 (13)	
C3	0.4897 (7)	0.6036 (5)	0.7740 (3)	0.0328 (14)	
C4	0.3955 (7)	0.7690 (5)	0.6888 (3)	0.0305 (14)	
C5	0.5421 (7)	0.4640 (5)	0.6637 (3)	0.0321 (14)	
C6	0.6979 (7)	0.6775 (4)	0.6743 (2)	0.0228 (11)	
C7	0.8265 (6)	0.6792 (4)	0.7293 (2)	0.0218 (11)	
C8	0.9276 (7)	0.7663 (5)	0.7384 (3)	0.0298 (13)	
H8	0.9138	0.8259	0.7105	0.036*	
C9	1.0477 (8)	0.7664 (5)	0.7879 (3)	0.0405 (16)	
H9	1.1132	0.8273	0.7950	0.049*	
C10	1.0734 (7)	0.6781 (6)	0.8273 (2)	0.0384 (16)	
H10	1.1578	0.6776	0.8606	0.046*	
C11	0.9740 (8)	0.5901 (5)	0.8175 (3)	0.0368 (15)	
H11	0.9918	0.5289	0.8439	0.044*	
C12	0.8507 (7)	0.5916 (5)	0.7701 (2)	0.0299 (13)	
H12	0.7812	0.5324	0.7649	0.036*	
C13	0.7818 (7)	0.9777 (4)	0.5270 (3)	0.0306 (13)	
H13	0.8715	1.0229	0.5316	0.037*	
C14	0.6886 (7)	0.9488 (5)	0.5760 (3)	0.0320 (13)	
H14	0.7068	0.9681	0.6199	0.038*	
C15	0.5638 (7)	0.8859 (4)	0.5475 (3)	0.0308 (13)	
H15	0.4799	0.8578	0.5685	0.037*	
C16	0.5844 (7)	0.8717 (5)	0.4830 (3)	0.0326 (14)	
H16	0.5187	0.8310	0.4528	0.039*	
C17	0.7188 (7)	0.9281 (5)	0.4710 (3)	0.0333 (14)	
H17	0.7605	0.9320	0.4309	0.040*	
C18	0.7079 (8)	0.6358 (5)	0.4693 (3)	0.0393 (16)	
H18	0.5976	0.6332	0.4569	0.047*	
C19	0.8207 (8)	0.6914 (5)	0.4373 (3)	0.0395 (16)	
H19	0.8003	0.7322	0.3992	0.047*	
C20	0.9681 (8)	0.6753 (6)	0.4722 (3)	0.0413 (16)	
H20	1.0653	0.7037	0.4618	0.050*	
C21	0.9484 (9)	0.6108 (5)	0.5246 (3)	0.0459 (18)	
H21	1.0292	0.5881	0.5563	0.055*	
C22	0.7886 (9)	0.5853 (5)	0.5224 (3)	0.0439 (17)	
H22	0.7425	0.5411	0.5520	0.053*	

### Atomic displacement parameters (Å^2^)

**Table:**

	*U*^11^	*U*^22^	*U*^33^	*U*^12^	*U*^13^	*U*^23^
Hf1	0.01979 (13)	0.02021 (13)	0.01715 (11)	0.00217 (9)	0.00150 (8)	0.00140 (8)
W1	0.02045 (13)	0.02513 (14)	0.02122 (12)	−0.00133 (9)	0.00071 (9)	0.00187 (8)
Cl1	0.0253 (8)	0.0370 (8)	0.0403 (7)	−0.0042 (7)	−0.0014 (6)	0.0026 (6)
O1	0.040 (3)	0.053 (3)	0.025 (2)	−0.007 (2)	−0.0049 (18)	0.0019 (19)
O2	0.025 (2)	0.066 (3)	0.065 (3)	−0.013 (2)	0.012 (2)	−0.007 (3)
O3	0.053 (3)	0.084 (4)	0.024 (2)	0.002 (3)	0.008 (2)	0.013 (2)
O4	0.067 (4)	0.036 (3)	0.052 (3)	0.016 (3)	−0.003 (2)	−0.011 (2)
O5	0.074 (4)	0.040 (3)	0.057 (3)	0.024 (3)	−0.020 (3)	−0.009 (2)
O6	0.028 (2)	0.024 (2)	0.0179 (16)	−0.0010 (17)	0.0020 (15)	0.0039 (14)
C1	0.017 (3)	0.023 (3)	0.034 (3)	−0.007 (2)	0.002 (2)	0.000 (2)
C2	0.033 (4)	0.033 (3)	0.031 (3)	0.000 (3)	0.006 (2)	−0.002 (3)
C3	0.028 (3)	0.040 (4)	0.030 (3)	−0.007 (3)	0.003 (2)	0.004 (3)
C4	0.028 (3)	0.038 (4)	0.025 (3)	0.001 (3)	0.001 (2)	−0.005 (3)
C5	0.026 (3)	0.039 (4)	0.029 (3)	0.003 (3)	−0.010 (2)	−0.001 (3)
C6	0.031 (3)	0.016 (3)	0.021 (2)	0.003 (2)	0.003 (2)	0.005 (2)
C7	0.017 (3)	0.027 (3)	0.022 (2)	0.005 (2)	0.002 (2)	−0.001 (2)
C8	0.023 (3)	0.034 (3)	0.032 (3)	−0.007 (3)	0.004 (2)	0.004 (3)
C9	0.036 (4)	0.051 (4)	0.034 (3)	−0.013 (3)	0.000 (3)	−0.005 (3)
C10	0.027 (3)	0.065 (5)	0.021 (3)	0.011 (3)	−0.004 (2)	−0.005 (3)
C11	0.032 (3)	0.050 (4)	0.027 (3)	0.007 (3)	0.000 (2)	0.005 (3)
C12	0.035 (3)	0.030 (3)	0.025 (3)	0.001 (3)	0.005 (2)	0.008 (2)
C13	0.028 (3)	0.014 (3)	0.051 (3)	−0.002 (2)	0.008 (3)	0.010 (2)
C14	0.037 (4)	0.026 (3)	0.033 (3)	0.005 (3)	0.005 (3)	0.003 (3)
C15	0.021 (3)	0.028 (3)	0.045 (3)	0.010 (2)	0.010 (3)	0.014 (3)
C16	0.029 (3)	0.030 (3)	0.036 (3)	0.006 (3)	−0.011 (3)	0.005 (2)
C17	0.029 (3)	0.038 (3)	0.033 (3)	0.006 (3)	0.005 (2)	0.019 (3)
C18	0.037 (4)	0.047 (4)	0.033 (3)	−0.009 (3)	0.004 (3)	−0.021 (3)
C19	0.047 (4)	0.049 (4)	0.023 (3)	0.004 (3)	0.005 (3)	−0.006 (3)
C20	0.030 (4)	0.050 (4)	0.046 (3)	0.010 (3)	0.012 (3)	−0.011 (3)
C21	0.055 (5)	0.038 (4)	0.043 (4)	0.018 (4)	0.002 (3)	−0.007 (3)
C22	0.071 (5)	0.022 (3)	0.041 (3)	−0.007 (3)	0.017 (3)	−0.013 (3)

### Geometric parameters (Å, °)

**Table:**

Hf1—O6	2.006 (3)	C8—H8	0.9500
Hf1—Cl1	2.4139 (14)	C9—C10	1.387 (9)
Hf1—C16	2.464 (5)	C9—H9	0.9500
Hf1—C17	2.465 (5)	C10—C11	1.395 (9)
Hf1—C18	2.469 (6)	C10—H10	0.9500
Hf1—C21	2.479 (6)	C11—C12	1.371 (8)
Hf1—C22	2.480 (6)	C11—H11	0.9500
Hf1—C20	2.483 (6)	C12—H12	0.9500
Hf1—C19	2.484 (5)	C13—C17	1.389 (8)
Hf1—C14	2.494 (6)	C13—C14	1.415 (8)
Hf1—C15	2.496 (5)	C13—H13	0.9500
Hf1—C13	2.514 (5)	C14—C15	1.408 (8)
W1—C2	2.019 (6)	C14—H14	0.9500
W1—C1	2.037 (6)	C15—C16	1.397 (8)
W1—C3	2.038 (6)	C15—H15	0.9500
W1—C4	2.040 (6)	C16—C17	1.395 (8)
W1—C5	2.044 (6)	C16—H16	0.9500
W1—C6	2.177 (6)	C17—H17	0.9500
O1—C1	1.137 (6)	C18—C22	1.401 (9)
O2—C2	1.156 (7)	C18—C19	1.417 (9)
O3—C3	1.135 (7)	C18—H18	0.9500
O4—C4	1.149 (7)	C19—C20	1.403 (9)
O5—C5	1.139 (7)	C19—H19	0.9500
O6—C6	1.291 (6)	C20—C21	1.393 (9)
C6—C7	1.508 (7)	C20—H20	0.9500
C7—C8	1.394 (8)	C21—C22	1.398 (10)
C7—C12	1.397 (7)	C21—H21	0.9500
C8—C9	1.381 (8)	C22—H22	0.9500
			
O6—Hf1—Cl1	97.55 (11)	O3—C3—W1	176.2 (6)
O6—Hf1—C16	108.77 (18)	O4—C4—W1	175.9 (6)
Cl1—Hf1—C16	132.40 (15)	O5—C5—W1	178.1 (6)
O6—Hf1—C17	133.27 (18)	O6—C6—C7	110.4 (5)
Cl1—Hf1—C17	101.53 (15)	O6—C6—W1	123.3 (4)
C16—Hf1—C17	32.89 (19)	C7—C6—W1	126.1 (4)
O6—Hf1—C18	100.41 (18)	C8—C7—C12	119.0 (5)
Cl1—Hf1—C18	134.01 (16)	C8—C7—C6	120.5 (5)
C16—Hf1—C18	79.9 (2)	C12—C7—C6	120.5 (5)
C17—Hf1—C18	96.2 (2)	C9—C8—C7	120.3 (6)
O6—Hf1—C21	91.47 (19)	C9—C8—H8	119.9
Cl1—Hf1—C21	83.03 (18)	C7—C8—H8	119.9
C16—Hf1—C21	133.2 (2)	C8—C9—C10	120.4 (6)
C17—Hf1—C21	132.8 (2)	C8—C9—H9	119.8
C18—Hf1—C21	54.7 (2)	C10—C9—H9	119.8
O6—Hf1—C22	78.04 (18)	C9—C10—C11	119.4 (5)
Cl1—Hf1—C22	114.19 (19)	C9—C10—H10	120.3
C16—Hf1—C22	109.7 (2)	C11—C10—H10	120.3
C17—Hf1—C22	129.0 (2)	C12—C11—C10	120.3 (6)
C18—Hf1—C22	32.9 (2)	C12—C11—H11	119.9
C21—Hf1—C22	32.7 (2)	C10—C11—H11	119.9
O6—Hf1—C20	124.08 (19)	C11—C12—C7	120.6 (6)
Cl1—Hf1—C20	80.31 (16)	C11—C12—H12	119.7
C16—Hf1—C20	113.1 (2)	C7—C12—H12	119.7
C17—Hf1—C20	101.1 (2)	C17—C13—C14	107.8 (5)
C18—Hf1—C20	54.6 (2)	C17—C13—Hf1	71.9 (3)
C21—Hf1—C20	32.6 (2)	C14—C13—Hf1	72.8 (3)
C22—Hf1—C20	54.1 (2)	C17—C13—H13	126.1
O6—Hf1—C19	131.56 (19)	C14—C13—H13	126.1
Cl1—Hf1—C19	109.27 (17)	Hf1—C13—H13	121.0
C16—Hf1—C19	82.0 (2)	C15—C14—C13	107.0 (5)
C17—Hf1—C19	80.5 (2)	C15—C14—Hf1	73.7 (3)
C18—Hf1—C19	33.3 (2)	C13—C14—Hf1	74.3 (3)
C21—Hf1—C19	54.5 (2)	C15—C14—H14	126.5
C22—Hf1—C19	54.5 (2)	C13—C14—H14	126.5
C20—Hf1—C19	32.8 (2)	Hf1—C14—H14	117.6
O6—Hf1—C14	82.99 (16)	C16—C15—C14	108.5 (5)
Cl1—Hf1—C14	91.77 (15)	C16—C15—Hf1	72.4 (3)
C16—Hf1—C14	54.65 (19)	C14—C15—Hf1	73.5 (3)
C17—Hf1—C14	54.37 (19)	C16—C15—H15	125.8
C18—Hf1—C14	132.1 (2)	C14—C15—H15	125.8
C21—Hf1—C14	171.9 (2)	Hf1—C15—H15	120.1
C22—Hf1—C14	149.4 (2)	C17—C16—C15	107.6 (5)
C20—Hf1—C14	152.4 (2)	C17—C16—Hf1	73.6 (3)
C19—Hf1—C14	133.5 (2)	C15—C16—Hf1	74.9 (3)
O6—Hf1—C15	80.05 (17)	C17—C16—H16	126.2
Cl1—Hf1—C15	124.55 (15)	C15—C16—H16	126.2
C16—Hf1—C15	32.70 (19)	Hf1—C16—H16	117.3
C17—Hf1—C15	54.02 (19)	C13—C17—C16	109.0 (5)
C18—Hf1—C15	100.2 (2)	C13—C17—Hf1	75.7 (3)
C21—Hf1—C15	151.8 (2)	C16—C17—Hf1	73.5 (3)
C22—Hf1—C15	119.2 (2)	C13—C17—H17	125.5
C20—Hf1—C15	145.3 (2)	C16—C17—H17	125.5
C19—Hf1—C15	112.9 (2)	Hf1—C17—H17	117.1
C14—Hf1—C15	32.78 (19)	C22—C18—C19	107.5 (6)
O6—Hf1—C13	114.49 (16)	C22—C18—Hf1	74.0 (3)
Cl1—Hf1—C13	79.09 (14)	C19—C18—Hf1	73.9 (3)
C16—Hf1—C13	54.2 (2)	C22—C18—H18	126.3
C17—Hf1—C13	32.39 (19)	C19—C18—H18	126.3
C18—Hf1—C13	128.5 (2)	Hf1—C18—H18	117.8
C21—Hf1—C13	150.0 (2)	C20—C19—C18	107.4 (6)
C22—Hf1—C13	161.3 (2)	C20—C19—Hf1	73.6 (3)
C20—Hf1—C13	119.7 (2)	C18—C19—Hf1	72.8 (3)
C19—Hf1—C13	109.8 (2)	C20—C19—H19	126.3
C14—Hf1—C13	32.82 (18)	C18—C19—H19	126.3
C15—Hf1—C13	53.89 (19)	Hf1—C19—H19	119.2
C2—W1—C1	87.3 (2)	C21—C20—C19	108.7 (6)
C2—W1—C3	88.5 (2)	C21—C20—Hf1	73.5 (4)
C1—W1—C3	173.9 (2)	C19—C20—Hf1	73.6 (3)
C2—W1—C4	93.7 (2)	C21—C20—H20	125.7
C1—W1—C4	88.9 (2)	C19—C20—H20	125.7
C3—W1—C4	86.9 (2)	Hf1—C20—H20	119.0
C2—W1—C5	90.3 (2)	C20—C21—C22	107.9 (6)
C1—W1—C5	90.7 (2)	C20—C21—Hf1	73.9 (4)
C3—W1—C5	93.8 (2)	C22—C21—Hf1	73.7 (4)
C4—W1—C5	175.9 (2)	C20—C21—H21	126.1
C2—W1—C6	179.2 (2)	C22—C21—H21	126.1
C1—W1—C6	92.1 (2)	Hf1—C21—H21	118.4
C3—W1—C6	92.1 (2)	C21—C22—C18	108.6 (6)
C4—W1—C6	85.8 (2)	C21—C22—Hf1	73.6 (4)
C5—W1—C6	90.1 (2)	C18—C22—Hf1	73.1 (4)
C6—O6—Hf1	171.4 (3)	C21—C22—H22	125.7
O1—C1—W1	174.9 (5)	C18—C22—H22	125.7
O2—C2—W1	177.5 (5)	Hf1—C22—H22	119.4
